# Spin state of a single-molecule magnet (SMM) creating long-range ordering on ferromagnetic layers of a magnetic tunnel junction – a Monte Carlo study†

**DOI:** 10.1039/d1ra05473b

**Published:** 2021-09-30

**Authors:** Andrew Grizzle, Christopher D'Angelo, José Martínez-Lillo, Pawan Tyagi

**Affiliations:** Center for Nanotechnology Research and Education, Mechanical Engineering, University of the District of Columbia Washington DC-20008 USA ptyagi@udc.edu; Instituto de Ciencia Molecular (ICMol), Universitat de València c/ Catedrático José Beltrán 2 Paterna València 46980 Spain

## Abstract

Paramagnetic single-molecule magnets (SMMs) interacting with the ferromagnetic electrodes of a magnetic tunnel junction (MTJ) produce a new system. The properties and future scope of new systems differ dramatically from the properties of isolated molecules and ferromagnets. However, it is unknown how far deep in the ferromagnetic electrode the impact of the paramagnetic molecule and ferromagnet interactions can travel for various levels of molecular spin states. Our prior experimental studies showed two types of paramagnetic SMMs, the hexanuclear Mn_6_ and octanuclear Fe–Ni molecular complexes, covalently bonded to ferromagnets produced unprecedented strong antiferromagnetic coupling between two ferromagnets at room temperature leading to a number of intriguing observations (P. Tyagi, *et al.*, *Org. Electron.*, 2019, **64**, 188–194. P. Tyagi, *et al.*, *RSC Adv.*, 2020, **10**, (22), 13006–13015). This paper reports a Monte Carlo Simulations (MCS) study focusing on the impact of the molecular spin state on a cross junction shaped MTJ based molecular spintronics device (MTJMSD). Our MCS study focused on the Heisenberg model of MTJMSD and investigated the impact of various molecular coupling strengths, thermal energy, and molecular spin states. To gauge the impact of the molecular spin state on the region of ferromagnetic electrodes, we examined the spatial distribution of molecule-ferromagnet correlated phases. Our MCS study shows that under a strong coupling regime, the molecular spin state should be ∼30% of the ferromagnetic electrode's atomic spins to create long-range correlated phases.

## Abstract 2

Paramagnetic single-molecule magnets (SMMs) interacting with the ferromagnetic electrodes of a magnetic tunnel junction (MTJ) produce new molecular spintronics testbed and highly ordered magnetic metamaterial promising for room temperature.

## Graphical abstract

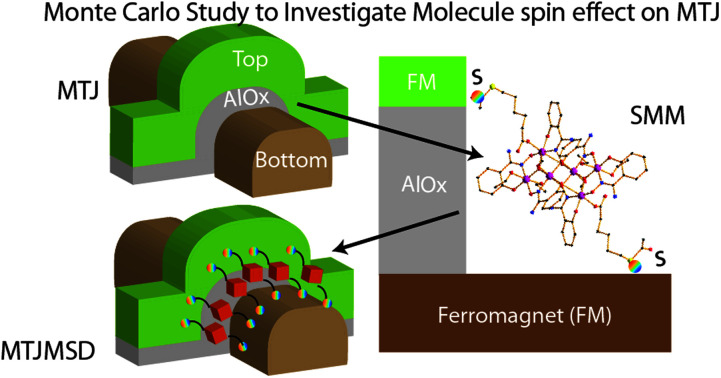

## I. Introduction

Molecules are the only mass-producible nanostructures with customizable chemical, electrical, optical, and magnetic properties that can be produced with sub-angstrom scale precision. Molecules are extremely versatile, and practically billions of types are possible, and so are the molecule-based devices.^1–3^ Several molecules such as single-molecule magnets (SMMs),^4^ porphyrin,^5^ DNA^6^ and organometallic molecules^7^ have a high potential to be included as the device element in future molecular spintronics devices (MSDs). MSD fabrication requires a molecule of interest to be simultaneously connected with at least a source and drain-type metal electrode.^8^ The intensity of interaction can be weak if it is physically separated from the two-metal electrode or connected by weak bonds.^9^ However, a molecule with functional groups like sulfur can form covalent and ionic bonds with metal electrodes leading to very strong coupling.^10,11^ In the strong coupling regime, molecules and metal electrodes near the interface show strong hybridization of energy levels.^12^ There exists a knowledge gap about the spin state of SMMs connected to metal electrodes. This paper focuses on investigating the effect of various levels of possible molecular spin states and their impact on MSDs. This study is expected to provide insights about the impact of a potential molecular spin state (*S*_m_) in the MSDs. The impact of *S*_m_ can be very different based on the level of molecular level hybridization with the metal electrodes. The strong hybridization between *S*_m_ and metal electrodes has been observed to create novel properties on both metal electrodes and molecules. For example, the interaction of thiolate molecule produced magnetism in a non-magnetic electrode^13^ and further enhanced the degree of spin polarization on ferromagnets. It is also well known that a molecule connected to metal electrodes cannot exhibit the properties measured in its isolated state. Therefore, the combined system of metal electrodes and molecules becomes a new composite system altogether.^13,14^ Understanding this system is extremely important to progress the field of MSDs, where SMM-like molecules possess a wide range of spin states interacting with magnetic electrodes.^13^ Magnetic electrodes, such as nickel (Ni), cobalt (Co), iron (Fe), exhibit strong long-range ordering. This long-range ordering can further transport the effect of molecule-ferromagnet interaction over the microscopic range. Our previous experimental studies showed that Mn hexanuclear^15^ and Fe–Ni octanuclear molecular complex (OMC)^14^ based SMMs produced long-range impacts on ferromagnetic electrodes leading to room temperature observations of several orders current suppression, spin photovoltaic effects, and several orders of magnitude magnetoresistance.^15,16^ Other groups have also observed strong coupling between C_60_ molecules and ferromagnetism of the nickel electrodes leading to the Kondo splitting phenomenon without applying the estimated ∼50 T field needed for this observation.^17^ However, experimentally determining the spin state of a paramagnetic molecule after forming a complete MSD is extremely challenging. Additionally, Density Function Theory (DFT) study is exceptionally challenging to simulate SMM-connected with a wide variety of long ferromagnetic electrodes of different shapes of MSDs at different temperatures.^18^

**Fig. 1:**
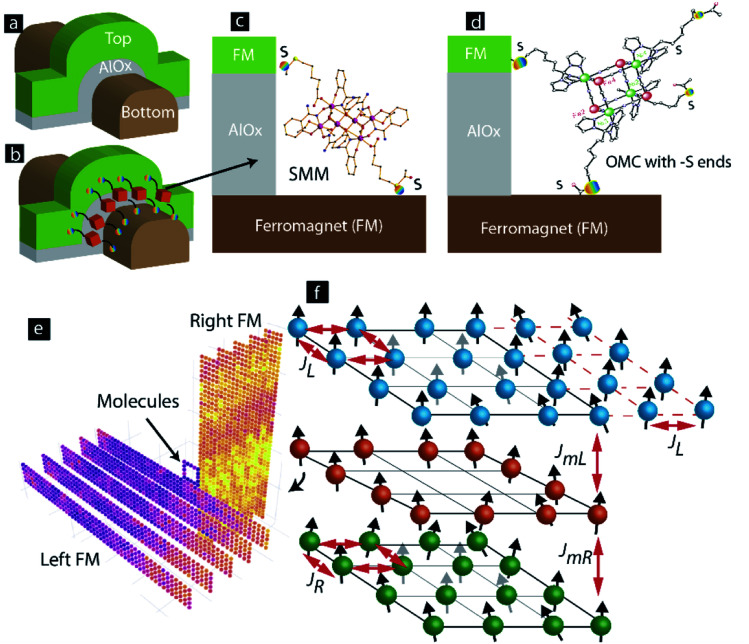
MSD formed by utilizing exposed edges of (a) a bare MTJ to attach (b) paramagnetic molecules between two ferromagnets. (c) SMM and (d) OMC paramagnetic molecules connected to ferromagnets *via* sulfur atom. (e) 3D Heisenberg model of molecular device. (f) Exchange coupling parameters associated with molecule-ferromagnet interactions.

This paper investigates the effect of molecular spin state on the experimentally studied cross junction-shaped MTJMSDs. MTJMSDs are experimentally studied to explore the intriguing phenomenon^15,16^ that arise when a bare magnetic tunnel junction (MTJ) (Fig. 1a) enables the stitching of paramagnetic molecular channels^2,19^ along the exposed edges (Fig. 1b). MTJMSD has been experimentally tested with two SMMs, the hexanuclear Mn_6_ (ref. 19) (Fig. 1c) and octanuclear Fe–Ni molecular complexes^2^ (Fig. 1d). The main difference between these two molecules is in the way atoms with a net spin state are connected *via* different chemistry, leading to different spin ground states. In the Mn_6_ based SMM, the magnetic exchange between Mn(iii) ions relies on the Mn–N–O–Mn torsion angles. These Mn_6_ molecules possessed *S* = 4 spin ground state. On the other hand, the OMC molecular complex exhibited *S* = 6 spin ground state due to strong exchange coupling between Fe and Ni *via* CN bridge (*i.e.*, Fe–C≡N–Ni). Extensive details about these two molecules are published elsewhere.^2,19^

When incorporated in an MTJMSD, both molecules produced unprecedented strong exchange coupling between ferromagnetic electrodes and current suppression at room temperature.^15,20^ It is noteworthy that OMC-produced current suppression was stable at room temperature,^20^ but the Mn_6_ based SMM yielded a transient current suppression.^15^ Interestingly, the core of SMM is connected to ferromagnetic electrodes with six atoms long alkane tethers. The core of OMC is connected to ferromagnetic electrodes with ten atom long alkane tethers. Since magnetic coupling decreases with the distance, the exchange coupling strength between ferromagnetic electrodes and SMM core (Fig. 1c) is expected to be more than the exchange coupling strength between the ferromagnetic electrodes and OMC core (Fig. 1d). It is noteworthy that the MTJMSD ground state is also a function of the magnitude of the molecular spin state (*S*_m_). It is important to note that a paramagnetic molecule connected to two metal electrodes will undergo Fermi level alignments. As a result, some charge transfer between molecule core and metal electrodes may occur. When connected to metallic electrodes, charge transfer between FM electrodes and molecules can produce new *S*_m_ spin states on the cores of the SMM and OMC.

Due to experimental challenges and limitations of DFT-like approaches, which generally work at zero temperature, the biggest knowledge gap is about the possible spin states of SMMs and their role on MTJMSD with extended ferromagnetic electrodes beyond the molecular junction area. To investigate the role of the molecular spin state, we have employed the Heisenberg Model^21^ of MTJMSD and conducted Monte Carlo Simulations (MCS). Since there is no verifiable way to measure exact *S*_m_ on SMM and OMC-like molecules in MTJMSDs, we have varied *S*_m_ over a range in the MCS studies. We have investigated the MTJMSD equilibrium properties as a function of *S*_m_ that may be correlated with the experimental observations. This approach enables us to cover a wide range of paramagnetic molecules without delving into their atomic structures. The selection of this approach is based on the successful application of MCS, explaining the experimental results obtained from MFM and SQUID magnetometry.^22^ This paper provides new insights into the effect of molecular spin state and evaluates the properties of the whole MTJMSD.

## II. Method

We have conducted the MCS study using an indigenously developed C++ program. We utilized a continuous spin model to allow spin vectors of the ferromagnets' atoms and molecules to assume any directions in a spherical coordinate system.^23^ To understand the property of experimentally studied MTJMSD *via* this MCS study, we focused on the Heisenberg model (Fig. 1e) as a 3D analog of an MTJMSD (Fig. 1b).^14^ This MCS study represented a tunnel barrier with empty space within a square-shaped molecular perimeter (Fig. 1f).^24^ In the MCS study, the exchange coupling parameter specific to the tunnel barrier was set to zero to simulate the case of the perfect tunnel barrier. With this provision, the MCS study discussed in this paper focused on the effect of paramagnetic molecule-induced impacts. An analysis of competing effects due to molecule and defect-induced exchange coupling was published elsewhere.^24^

In general, in this MCS study, two FM electrodes possessed five atom width, five atom thickness, and 50 atom length, unless stated otherwise. It is noteworthy that the dimension of length, width, and height is described in terms of the number of atoms fitted along each physical dimension (Fig. 1e and f). This approach of defining physical dimensions is consistent with prevalent convention^23^ and our prior MCS study that yielded valuable insights related to experimental observations on MTJMSDs.^14^

**Fig. 2:**
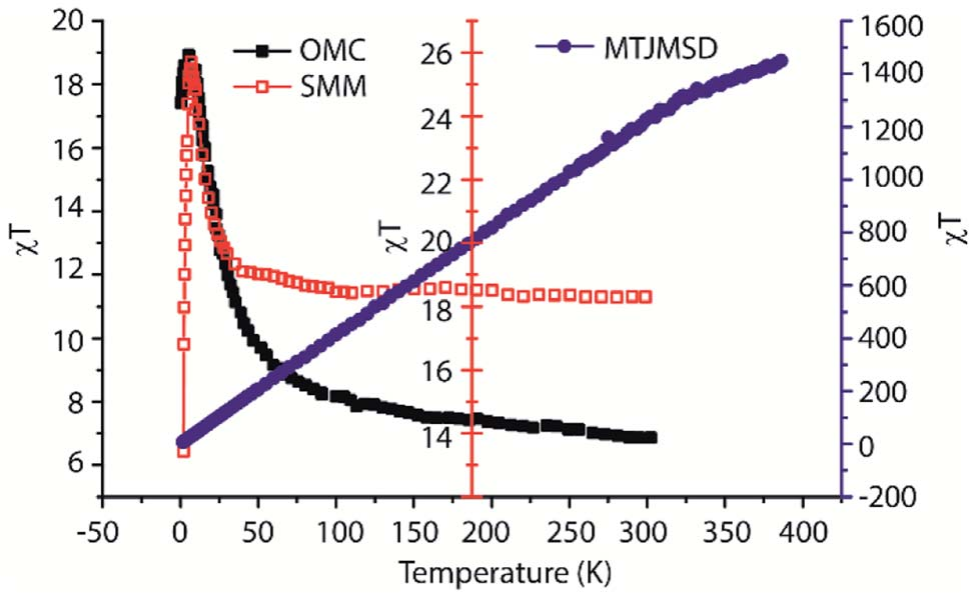
*χT vs. T* plot for OMC and SMM molecules. The plot for MTJMSD followed different trend as compared to molecules. OMC data adopted from ref. 7.

For representing molecules along the edges of an MTJ, a square-shaped molecular ring was introduced at the cross junction of two FM electrodes (Fig. 1e). The perimeter of the molecular ring was a 5 × 5 square with 16 molecular analogs fitting in it. Paramagnetic SMM molecules of MTJMSD (Fig. 1d) were represented by the atomic scale analog with adjustable spin (*S*_m_) parameter. The rationale for representing complex SMM molecules with the atomic analog is the following: (i) prior molecular device research has successfully employed generic analytical models to understand experimental data. For example, Simmons tunneling model^25^ was used to understand the transport characteristics through SMMs.^11,15,20^ (ii) Molecules in the device form generally follow generic single-electron device physics.^26^ (iii) According to experimental data on powder form, SMMs generally settle in different spin states at different temperatures. Such tendency is clearly observed in *χT vs. T* plots (Fig. 2). For example, isolated OMC molecules changed spin state from 6 to 3 when the temperature was increased from 2 to 60 K.^2^ However, when connected to ferromagnetic electrodes in MTJMSD, the whole assembly *χT vs. T* was radically different (Fig. 2). To maximize the impact of SMM and to produce measurable signals, the MTJMSD used in Fig. 2 was fabricated in the form of pillars. For this study, ∼7000 MTJMSD were produced on a chip where the two FM electrodes' dimensions and the insulator were the same and OMCs were bridged across the insulator gap along the exposed side edges. Experimental details of sample fabrication are published elsewhere.^14^ It is extremely challenging to experimentally determine the exact molecular spin state in MTJMSD. Therefore, we have parametrically varied the spin state of a molecular analog (Fig. 1e and f) to investigate the impact of various molecular spin states without delving into the simulation of complex molecular structures (Fig. 1c and d). Extensive experimental details about these molecules and MTJMSD are published elsewhere.^2,14,19^

In the MCS study, the coupling between two FM electrodes occurred by the paramagnetic molecules (Fig. 1f). The molecule-mediated exchange coupling between the left and right FM electrodes is governed by the two molecule's coupling parameters with the left electrode (*J*_mL_) and molecule coupling with the right electrode (*J*_mR_), respectively. The positive and negative signs of *J*_mL_ and *J*_mR_ governed whether molecule made ferromagnetic and antiferromagnetic coupling with FM electrodes. The magnitude of *J*_mL_ and *J*_mR_ covers the strength of exchange coupling between molecules and ferromagnets. It is noteworthy that variation of *J*_mL_ and *J*_mR_ covers several possible scenarios arising due to the use of different lengths of molecular tethers utilized to connect molecular core with metal electrodes (Fig. 1d). A dedicated study focusing on *J*_mL_ and *J*_mR_ has been published elsewhere.^22^ The MTJMSD energy with different parameters was calculated using eqn (1). To simulate the effect of change in temperature, we varied thermal energy (*kT*) of the MTJMSD Heisenberg model in energy (*U*) eqn (1).1
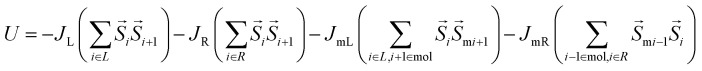


In this study, *S* is a 3D vector that represents the discrete atomic spin of FM electrodes. *S*_m*i*_ vectors represent the *S*_m_ of molecules at *i*^th^ position. *S*_m_ was varied over the 0 to 4 range. However, the main discussion is around the critical *S*_m_ values for which transition in the molecular device was observed. *J*_L_, and *J*_R_, are the Heisenberg exchange coupling strengths for the left and right FM electrodes (Fig. 1b). In our MCSs, the atoms beyond the boundary of the MTJMSD model (Fig. 1b) were set with zero spin state.^23^ The energy (*U*), described in eqn (1), of the whole system was minimized by running the Markov chain process. Markov process led to a stable low energy state. Further details of MCS are published elsewhere.^14^ MCS study was started with an initial state where each atom and molecule's spin vector were randomly oriented. To reaching the equilibrium state, a new spin state was created on randomly selected molecules and FM layers. To produce a new spin state, we only varied spin vector direction in 360° in 3D during each step of the simulation. New spin states were selected or rejected according to the Metropolis algorithm.^23^ If the energy of MTJMSD decreased with the new spin vector at a site was accepted. However, if the energy of the MTJMSD increased with respect to initial energy, then a new spin vector was accepted based on the criteria represented in eqn (2).^23^

2 exp(−(Δ*U*/*kT*) ≥ *r*

**Fig. 3:**
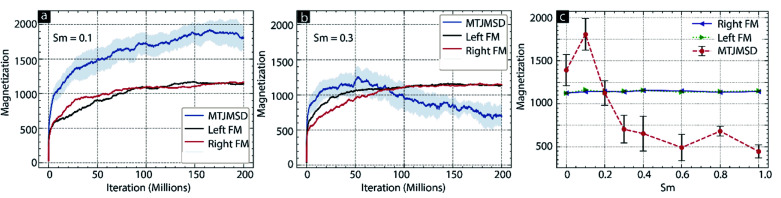
Iteration count *vs.* magnetic moment of MTJMSD, left FM, and right FM for (a) *S*_m_ = 0.1, (b) *S*_m_ = 0.3, (c) MTJMSD and FM electrode magnetic moment for molecular spin ranging 0 to 1. For all the cases *kT* = 0.1, *J*_mL_ = −1 and *J*_mR_ = 1.

Hence, if the left side of the eqn (2) was more than a random number (*r*), generated between 0 and 1, the new spin state was also accepted. This process occurred 200–2000 million times to yield the equilibrium MTJMSD states. The evolution of MTJMSD magnetic moment with increasing iteration count is plotted in Fig. 3. After each simulation study, we obtained the final MTJMSD with the equilibrium spin orientation information as a 3D lattice plot. Simulated 3D lattice plots were unable to present the numerical value of the spatial correlation between the molecule spin state and the different regions of FM-electrodes. We computed the dot product between molecular spin and the average of atomic spins in each row (along the width) for each FM layer to represent the numerical value of correlation factor (*c*). The equation for computing the spatial correlation factor (*c*) is mentioned below.

3 *c* = (*S*_m_*x⃑* + *S*_m_*y⃑* + *S*_m_*z⃑*) × (*S*_FM_*x⃑* + *S*_FM_*y⃑* + *S*_FM_*z⃑*)

The *c* = 1 suggests a strong correlation and parallel alignment of molecules' and ferromagnet spins. The *c* = −1 magnitude of the correlation factor represents strong correlation and antiparallel alignment between molecules and the FM electrode atoms. The magnitude of *c* varies between −1 to 1. Here −1 demonstrates a strong antiferromagnetic correlation while +1 shows a strong ferromagnetic correlation between the average magnetic moment of molecules and the magnetic moment of individual atoms of the two FM electrodes of the MTJMSD's Heisenberg model.

The units of total energy *U* and exchange coupling parameters are the same as *kT*. In this study, the exchange coupling parameters and *kT* are referred to as the unitless parameters. The overall magnetic moment of the MTJMSD is the sum of the magnetic moment of the molecules, left FM and right FM electrodes. The magnetic moment in MCS is defined as the sum of spin vectors for a region and represented as the unitless parameter and consistent with the conventional definition of magnetic moment in MCS.^23^ We have mainly focused on the molecule-induced strong antiferromagnetic coupling where *J*_mL_ = −1 and *J*_mR_ = 1. The reason for the emphasis on molecule-induced antiferromagnetic coupling is the observation of molecule-induced strong exchange coupling in our prior experimental work.^14^ We also varied molecular coupling strength, *kT*, molecular spin state, and MTJMSD dimensions to make this study generic.

## III. Results and discussions

First, we studied the impact of molecular spin state (*S*_m_) on the temporal evolution of MTJMSD and focused on the case of molecule-induced strong antiferromagnetic coupling (*J*_mL_ = −1 and *J*_mR_ = 1). According to our previous study, OMC induced strong antiferromagnetic coupling.^14^ Since we experimentally observed molecule-induced strong antiferromagnetic coupling well above room temperature,^14^ we have investigated MTJMSD temporal evolution at *kT* = 0.1. To investigate the impact of *S*_m_, we recorded the magnetic moment of the MTJMSD and its different components as a function of iterations steps; it is noteworthy that iteration steps are equivalent to the time dimension. We generally ran an MCS over ∼200 million iterations and recorded the magnetic moment of the FM electrodes, molecules, and whole MTJMSD at the interval of 50 000 steps. We varied *S*_m_ from 0 to 4 range. However, we observed that the nature of MTJMSD stabilization dramatically changed between *S*_m_ = ∼0.1 (Fig. 3a) and *S*_m_ = ∼0.3 (Fig. 3b). For *S*_m_ > 0.3 MTJMSD stabilized in a similar manner. For *S*_m_ ≤ 0.1, the magnetic moment of the left ferromagnet (Left-FM) and right ferromagnet (Right-FM) stabilized around 1200 (ESI-Fig. S1†). For this case, the overall magnetic moment of MTJMSD was around 1400. However, MTJMSD with *S*_m_ = 0.1 stabilized near 2000 (Fig. 3a). Increasing *S*_m_ from 0 to 0.1 produced parallel alignment of FM electrodes even though the nature of molecular coupling (*J*_mL_ = −1 and *J*_mR_ = 1) had a tendency to promote antiparallel alignment of FM electrodes. For *S*_m_ ≥ 0.3, left-FM and right-FM both still stabilized around 1000. However, MTJMSD's total magnetic moment, which is the sum of the magnetic moment of left-FM, right-FM, and molecules, started settling below the individual electrode magnetic moment around 600. This result suggests that even though the molecule made the same level of strong coupling with two electrodes but, *S*_m_ dictate the MTJMSD stabilization dynamics. We also explored the effect of a wider range of *S*_m_ (Fig. 2c) on MTJMSD and left and right FM electrodes. The left-FM and right FM electrodes settled around 1100, *i.e.*, close to their maximum possible magnetic moment of FM electrodes, *i.e.*,1250 for *S*_m_ range from 0 to 1 (Fig. 3c) and 0–4 range (ESI-Fig. S2†). Interestingly, around *S*_m_ = 0.2, the molecule started forcing left-FM and right-FM to settle in the antiparallel state due to the molecule-induced antiferromagnetic coupling (Fig. 3c). This result suggests that strong exchange coupling between molecule and FM electrodes can only impact MTJMSD when the molecular spin magnitude is above a critical value, *i.e.*, *S*_m_ = 0.2. For *S*_m_ = 4, we saw FM electrode, and MTJMSD stabilization pattern was similar to that of *S*_m_ = 1 (ESI-Fig. S2†). However, the major difference was that the MTJMSD magnetic moment became lower than that of left-FM and right-FM electrodes from a very early stage. It means increasing *S*_m_ promoted early stabilization of MTJMSD into an antiferromagnetic state.

Based on the simulation results providing the *S*_m_ limit required to observe the long-range effects (Fig. 3c) at the high temperature, we can deduce that molecular spin state in our prior experimental work at room temperature.^15,20^ In previous work, we observed that OMC produced stable current suppression at room temperature.^20^ However, Mn_6_ SMM produced transient current suppression at room temperature.^15^ Different experimental responses from two types of paramagnetic molecules suggest that OMC might have attained higher *S*_m_ > 0.2. Whereas Mn_6_ SMM appears to attain *S*_m_ value around 0.2, assuming ferromagnetic electrodes did not yield a significant impact. We also experimentally observed that MTJMSD settled in multiple metastable states for several days.^15,20^ It means the molecular spin state is expected to fluctuate around a critical value, and simulation study in this paper suggests that critical value is ∼0.2 (Fig. 3c).

**Fig. 4:**
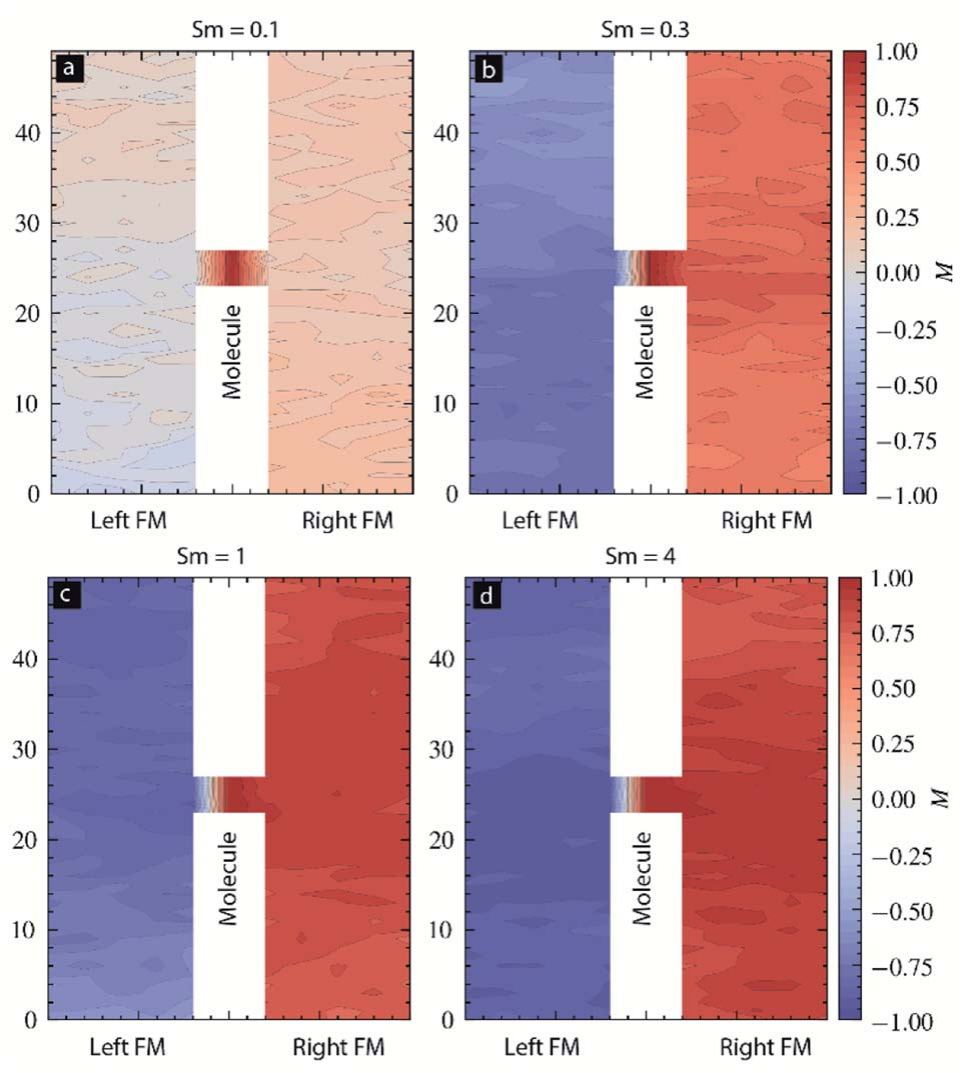
Spatial distribution of molecular spin correlation factor for molecular spin (a) 0.1, (b) 0.3, (c) 1.0, and (d) 4.0. For all the cases *kT* = 0.1, *J*_mL_ = −1 and *J*_mR_ = 1.

The temporal evolution discussed in Fig. 3 did not provide any details about the spatial impact range of *S*_m_ along the physical dimensions of each electrode. Understanding the spatial range is critical in understanding how far a molecule's *S*_m_ influence can penetrate along the length and thickness of FM electrodes. To calculate the spatial correlation between molecular spin state and the magnetic electrode's spin state, we calculated the dot product between the average magnetic moment of the molecules with each atom's magnetic moment in left-FM and right-FM and termed this product as correlation factor (*c*). We studied the correlation factor for each molecular spin state covered in this MCS study. To make discussion focused around critical *S*_m_ we mainly focused on selected values. For *S*_m_ = 0.1, correlation factor was in −0.25 to 0.25 range (Fig. 4a). This poor correlation between FM electrodes and *S*_m_ is consistent with the temporal evolution graph observed for *S*_m_ = 0.1 (Fig. 3a); *S*_m_ < 0.2 could not direct the FM electrodes according to the nature of molecular coupling with the two electrodes. However, for *S*_m_ = 0.3 stronger correlation factor was observed for each FM layer. The molecule magnetic alignment with respect to left-FM and right-FM electrodes was antiparallel and parallel, respectively (Fig. 4b). The magnitude of the correlation factor was around 0.5 on both electrodes (Fig. 4b). Interestingly, for *S*_m_ = 0.3, a spatial correlation on the left electrode was non-uniform along the electrode length and width (Fig. 4b). The correlation factor toward the top and bottom end approached near 0 (uncorrelated) and −1 (highly correlated) (Fig. 4b). Due to the influence of molecular spin, the left FM electrode is expected to behave very differently. For instance, injection of up-direction spin-polarized electrons may face high resistance when injected from the lower end of the left electrode. However, for the same type of spin, injection resistance is expected to be much lower. Interestingly, the correlation factor of the right FM electrode for *S*_m_ = 0.3 is relatively low towards the end (∼0.5) and high near the molecular junction (∼0.75) (Fig. 4b). The implication of such a difference in molecule correlated phases on the right electrode is expected to produce different resistance for the electron flow. However, our current MCS program is unable to compute resistance as a material property. It is noteworthy that correlated phases shown in Fig. 4b are not expected to be exactly reproducible. It is because of the reason that each MCS study involves random selection of atoms and random creation of spin vectors as an MTJMSD evolves into an equilibrium state. Since near *S*_m_ = 0.3 MTJMSD may exhibit several metastable states, MCS may stabilize into slightly different phases at the end of each study. Different phases in FM electrodes and molecular spin states may differ in correlated phases from simulation to simulation, although trends are consistent over several studies with identical parameters.

For *S*_m_ = 1, the trend was comparable to *S*_m_ = 0.3 cases, except the correlation factor became higher and was ∼0.75 (Fig. 4c). For *S*_m_ = 4, the trend was comparable for *S*_m_ = 0.3–1 case, and the correlation factor became more intense, reaching close to 1 (Fig. 4d). The intensity of the correlation factor near the molecular junction increased beyond the level observed for lower spins (Fig. 4d). In summary, left-FM-molecule-right-FM appears as a single highly correlated system for *S*_m_ ≥ 0.2 (Fig. 3c and 4). The spatial correlation suggests that the magnetic electrode must be strongly influenced near the MTJMSD junction.

**Fig. 5:**
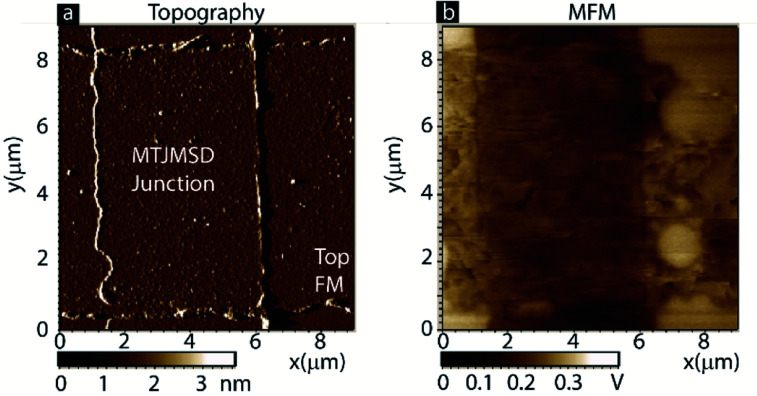
(a) Topography and (b) MFM of an MTJMSD. MFM of top electrode showing development of different magnetic phases due to molecule induced coupling.

To verify this MCS data, we have conducted an experimental magnetic force microscopy (MFM) study on an MTJMSD. Cross junction shaped MTJMSD was formed from an MTJ of Ta/Co (5–7 nm)/NiFe (5–3 nm)/AlO*x* (∼2 nm)/NiFe (10 nm) thin-film configurations and OMC paramagnetic molecules. The 3D device structure is shown in Fig. 1b. A zoomed-in view of the OMC and ferromagnetic electrode interaction along the exposed side edges is shown in Fig. 1c. Indeed, we have observed MTJMSD, which appears physically intact (Fig. 5a), showed intriguing new phases around the junction area (Fig. 5b).

It is noteworthy that this particular MTJMSD exhibited molecule-induced strong antiferromagnetic coupling at room temperature.^14^ Hence the MCS data shown in Fig. 4 for *kT* = 0.1 and strong molecule induced antiferromagnetic coupling (*J*_mL_ = −1 and *J*_mR_ = 1) is a good representation of the experimental data shown in Fig. 5. We have experimentally observed many molecule correlated phases in MFM imaging at room temperature.^16^ The in-depth discussion of the experimental details about the fabrication and MFM experiments are published elsewhere.^14,16^ Based on the simulation results reported in this paper, we estimated that the OMC spin state in the experimentally produced MTJMSD is well above 0.3 at room temperature. Since MTJMSD demonstrated multiple magnetic phases around tunnel junctions, we estimated that molecular spin states might differ for different magnetic phases.

We also investigated the spatial magnetic susceptibility of MTJMSD. For the molecule-specific magnetic susceptibility calculation, the magnetic moment of 16 molecules was utilized. However, for the calculation of spatial magnetic susceptibility of the FM electrodes, the magnetic moment (*m*) of each atom present along the width dimensions, shorter dimension parallel to the molecular plane, of each FM electrode were utilized (eqn (4))^23^

4 *χ* = *kT* × *N*(〈*m*^2^〉 − 〈*m*〉^2^)

**Fig. 6:**
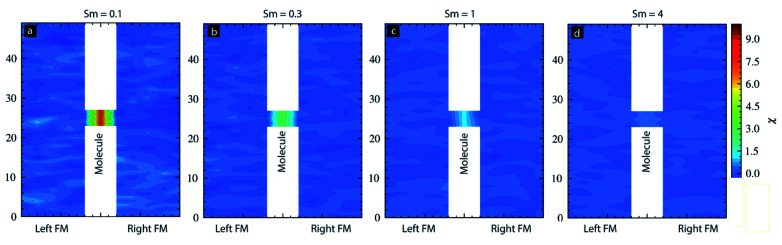
Magnetic susceptibility (*χ*) of FM electrodes and molecular layers of MTJMSD for (a) *S*_m_ = 0.1, (b) *S*_m_ = 0.3, (c) *S*_m_ = 1, and (d) *S*_m_ = 4. For all the cases *kT* = 0.1, *J*_mL_ = −1 and *J*_mR_ = 1.

For the case of *S*_m_ = 0.1, molecules' magnetic susceptibility (*χ*) was very high as compared to the two FM electrodes (Fig. 6a). A higher *χ* for molecule suggests that for *S*_m_ = 0.1, the external magnetic field can align the molecular spin vector selectively. However, for *S*_m_ = 0.3 cases, the magnitude of *χ* for molecular and ferromagnetic electrode regions was around 4 and 0, respectively (Fig. 6b). For *S*_m_ = 1, this difference between the *χ* for molecules and magnetic electrode were ∼1 and 0, respectively (Fig. 6c). Ultimately, for *S*_m_ = 4, the value of *χ* for molecules and FM electrodes was almost the same and near 0 (Fig. 6d). This study suggests that if an MTJMSD possesses strongly exchange-coupled high spin molecular magnets, then realizing selective switching of molecules will be highly challenging.

**Fig. 7:**
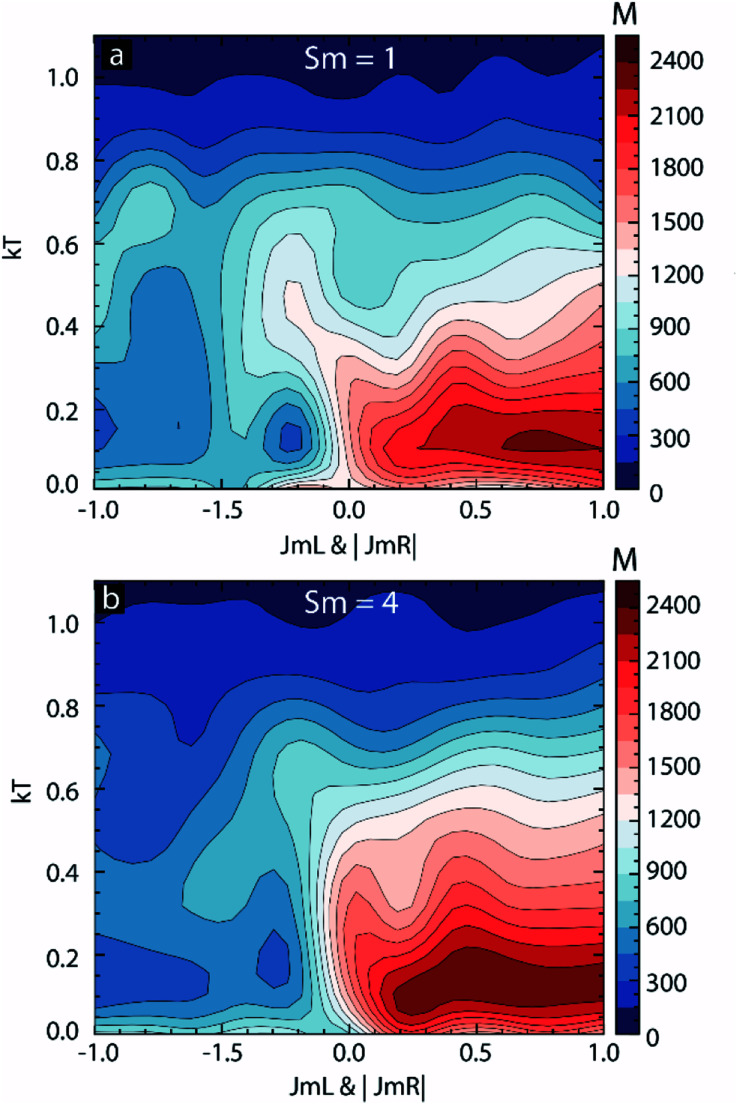
Contour plots of magnetic moment of MTJMSD as a function of *kT* and *J*_mL_ & |*J*_mR_| for (a) *S*_m_ = 1, and (b) *S*_m_ = 4.

In this paper the data discussed in Fig. 2–4 we only limited to *kT* = 0.1 and *J*_mL_ = −1 and *J*_mR_ = 1. To make this study applicable for a wide range of possibilities, we investigated the effect of thermal energy and molecular coupling strengths on MTJMSDs for different *S*_m_. To investigate the effect of thermal energy, we varied *kT* from 0.01 to 1.1. The molecular coupling strength was varied by ensuring that the modulus of *J*_mL_ and *J*_mR_ were equal. The *J*_mR_ was positive while *J*_mL_ was swept from −1 to 1. We simulated the full range by this approach when the molecule could induce antiferromagnetic to the ferromagnetic coupling of varying strengths. The simultaneous variation in molecule-FM electrode coupling in Fig. 7 is shown by *J*_mR_ = |*J*_mL_|. The contour plot for *S*_m_ = 0 shows that MTJMSD's magnetic moment settled in high and low magnitude state irrespective of the sign and magnitude of *J*_mL_ & |*J*_mR_| (ESI-Fig. S4†). Increasing *kT* settled MTJMSD into a highly disordered state producing a low MTJMSD magnetic moment. Contour plot for *S*_m_ = 1 and *kT* < 0.2 the MTJMSD's magnetic moment remained close to 300–900 for −1 to −0.2 range of *J*_mL_ & |*J*_mR_| (Fig. 7a). Interestingly, a relatively low magnetic moment state was more prevalent around the *J*_mL_ & |*J*_mR_| = −0.5. These results suggest that MTJMSD overall magnetic phases do not change monotonically with *J*_mL_ & |*J*_mR_| and *kT*.

As *kT* increased, the MTJMSD started attaining the higher magnetic moment and finally settled into a low magnetic moment state due to thermal energy-induced disordering (Fig. 7a). Contour plot for *S*_m_ = 1 and *kT* < 0.2 the MTJMSD's magnetic moment was as high as ∼2400 for positive *J*_mL_ & |*J*_mR_| (Fig. 7a). For a positive sign of *J*_mL_ and *J*_mR_, as *kT* increased, the MTJMSD's magnetic moment started attaining the lower magnetic moment and finally settled into a low magnetic moment state due to thermal energy induced disordering (Fig. 7a). However, the highest MTJMSD magnetic moment state appeared for *J*_mL_ & |*J*_mR_| > 0.6. The contour plot for *S*_m_ = 4 was somewhat similar to that of *S*_m_ = 1 (Fig. 7b). However, for *S*_m_ = 4 and *kT* < 0.2, the MTJMSD's magnetic moment persisted around ∼500 for weaker molecular coupling. For instance, MTJMSD magnetic moment state that was seen for *S*_m_ = 1 around *kT* = 0.1–0.2 for *J*_mL_ & |*J*_mR_| ≤ −0.9 was seen for *S*_m_ = 4 around *kT* = 0.1–0.2 for *J*_mL_ & |*J*_mR_| ≤ −0.6 (Fig. 7b). Also, MTJMSD magnetic moment state that was seen for *S*_m_ = 1 around *kT* = 0.1–0.2 for over very tight space for positive *J*_mL_ (0.6–1) was seen over a broad range for *S*_m_ = 4 around for 0.2 ≤ *J*_mL_ & |*J*_mR_| ≤ 1 (Fig. 7b). Hence, *S*_m_ played an important role in deciding the overall MTJMSD magnetic moment. The variation in *S*_m_ and its impact on MTJMSD are expected to appear in the form of experimentally observed several orders of magnitude conductivity changes.^16,27^

**Fig. 8:**
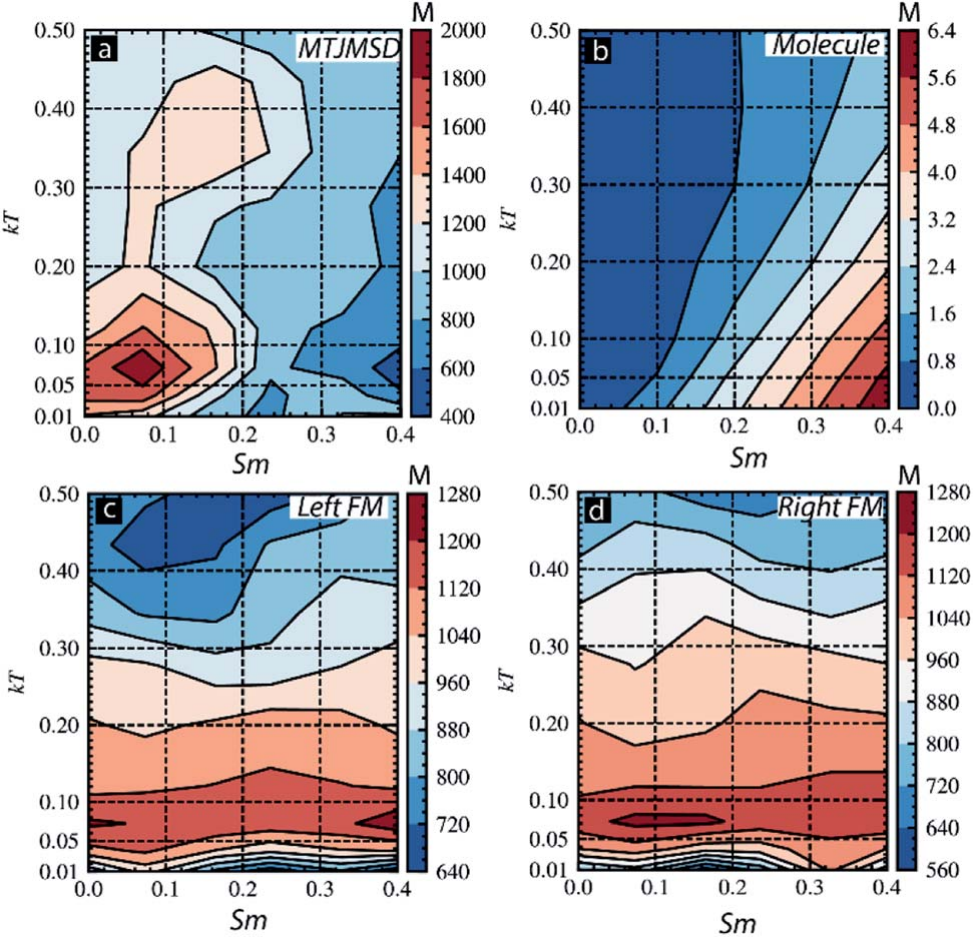
Contour plot showing magnetic moment for thermal energy (*kT*) and *S*_m_ for (a) full MTJMSD, (b) molecular layer, (c) left-FM, and (d) right-FM. For all the cases molecular coupling was *J*_mL_ = −1 and *J*_mR_ = 1.

We also investigated the effect of *S*_m_ and thermal energy on various parts of the MTJMSDs (Fig. 8). For this study, we focused on *S*_m_ ranging from 0 to 0.4 and *kT* ranging from 0.01 to 0.5 for *J*_mL_ = −1 and *J*_mR_ = 1. The ranges of *S*_m_ and *kT* is selected to focus on major transitions observed in Fig. 3c and 7. In the contour plot of MTJMSD's magnetic moment was ∼2000 for *S*_m_ < 0.2 and *kT* < 0.1 (Fig. 8a). However, as *S*_m_ goes beyond 0.2, MTJMSD started to settle in the low magnetic moment state due to molecule-induced strong antiferromagnetic coupling (Fig. 8a). This result is congruent with the data shown in Fig. 3c. It is important to note that with increasing *kT*, for *S*_m_ < 0.2, MTJMSD loses a high magnetic moment state very rapidly as compared to the variations observed for *S*_m_ > 0.2 (Fig. 8a). It is apparent that MTJMSD magnetic moment starts to get coupled with the molecular spin state for *S*_m_ > 0.2, which remains stable for higher thermal energy. The molecule's cumulative magnetic moment also gets impacted due to *kT* (Fig. 8b). The net magnetic moment of the molecule got disturbed with a slight increase in *kT* (Fig. 8b). However, as *kT* increases, the molecular magnetic moment persisted more for the higher magnitude of *S*_m_. However, left-FM (Fig. 8c) and right-FM (Fig. 8d) both showed high magnetic moment for *kT* < 0.2 over 0–0.4 molecular spin magnitude. Electrode finally settled into a thermally induced disturbed low magnetic moment state (Fig. 8c and d). The main message this study suggests is that uniform molecular magnetic moment existed around linear boundaries on *S*_m_*vs. kT* graph (Fig. 8b).

**Fig. 9:**
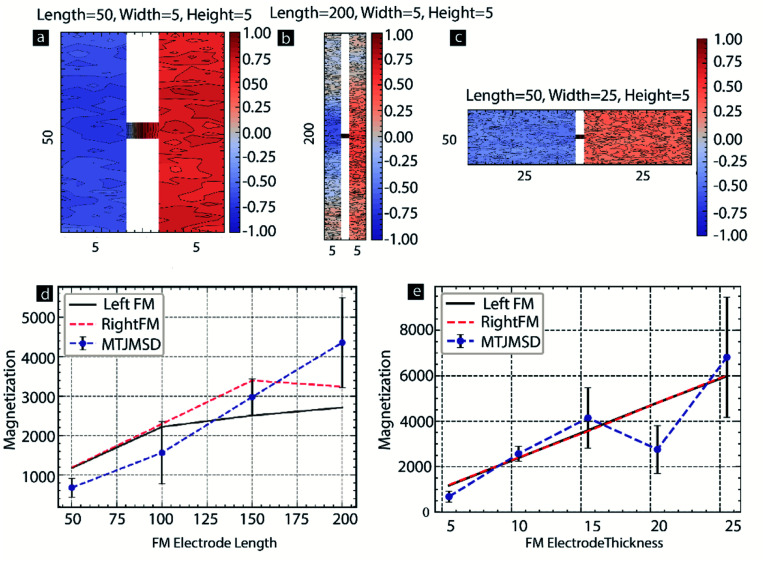
Spatial correlation factor for FM electrodes with (a) length = 50, width = 5, and height = 5 (b) length = 200, width = 5, and height = 5, (c) length = 50, width = 25, and height = 5. (d) Magnetization *vs.* FM electrode length (e) magnetization *vs.* FM electrode thickness. For all the cases *S*_m_ = 0.2. *kT* = 0.1, *J*_mL_ = −1 and *J*_mR_ = 1. Here, width = thickness of FM electrodes.

We also investigated the effect of MTJMSD's dimensions along with *S*_m_. For this study, we changed the length of the left-FM and right-FM electrodes from 50 to 200, keeping the width and height to 5. We utilized the correlation factor as the parameter to investigate the effect of MTJMSD dimensions. Discussion about the computation of correlation factors is discussed elsewhere in this paper in the context of Fig. 4. The analysis of the spatial correlation factor indicated that for MTJMSD of 50 atom length, the molecules were strongly correlated with the magnetic moment of the left-FM and right-FM (Fig. 9a). However, for 200 atomic length MTJMSDs, molecules were only correlated to the FM electrodes near the junction area (Fig. 9b). Similarly, we also increase the thickness of each FM electrode from 5 to 25, while the length and width were fixed to 50 and 5, respectively. Spatial correlation data for the extreme case of thickness = 25 suggest that left-FM and right-FM electrodes were weakly correlated with the molecules' magnetic moment. However, unlike 200 atomic length MTJMSD, the spatial correlation factor was relatively uniform over the whole MTJMSD for 50 atoms thick MTJMSD (Fig. 9c). For further investigation, we plotted the magnetic moment of the MTJMSD and two FM electrodes as a function of the electrode length (Fig. 9d). The effect of molecule-induced strong exchange coupling could force the large area of MTJMSD only for short lengths (Fig. 9d). As length doubled, MTMSD's left-FM and right FM electrode stop aligning perfectly antiparallel to each other, and many metastable phases started becoming possible. As length increased to 150, the MTJMSD magnetic moment was in between the left-FM and right-FM electrodes (Fig. 2c). It is apparent that as the length of the electrode increases to 150 or more, FM electrodes appear to have multiple phases leading to lowered magnetic moment (Fig. 9d). Since increasing length did not allow the antiparallel alignment of the two FM electrodes over the entire length, MTJMSD's net magnetic moment was significantly high. The increase in thickness of the FM electrode was more influential in determining the *S*_m_ effect on MTJMSD (Fig. 9e). Generally, increasing thickness forced MTJMSD to settle in a higher magnetization state above the individual FM electrode's magnetic moment (Fig. 9e). Each data point in Fig. 9d and e was repeated five times, and simulations were conducted for 2 billion iterations to ensure we reached an equilibrium state.

Interestingly, for the 20-atom thick FM electrode thickness, the MTJMSD's magnetic moment was consistently below the FM electrode magnetic moment. We are unsure about the actual mechanism of why 20-atom thick FM electrode-based MTJMSD were different than those of 15 and 25 atom thick FM electrodes. We hypothesized that changing the dimensions of the FM electrode impacted the stabilization dynamics; for the 20-atom thick FM electrode thickness, the equilibrium magnetization state was akin to the 5 atoms thick FM electrode, promoting antiparallel alignment of the two ferromagnetic electrodes (Fig. 9e). It appears that 20 atom thick FM electrode-based MTJMSD cancels the magnetic moment of one FM electrode due to the antiparallel alignment of the second electrode. As a result, the net magnetic moment of the MTJMSD was lower than the magnetic moment of the individual FM electrodes possessing similar order of magnetic moment (Fig. 9e). The size effect data shown in Fig. 9 provide direct insights into the consequences of varying the FM electrode dimensions.

The effect of molecular spin state strongly coupled to the FM electrodes was observed in several published studies by our group,^14,16,20,27,28^ and others.^17^ Unfortunately, it is extremely challenging to measure the exact molecular spin state connected between two ferromagnetic electrodes along the vertical multilayer edge of a tunnel junction (Fig. 1c and d). Hence, we cannot directly compare SMM spin states calculated in this paper and prior experimental studies. However, several studies provide direct evidence that molecular spin state, when strongly coupled to ferromagnetic electrodes, created a new phenomenon that could not be seen when SMMs were connected to non-magnetic electrodes or magnetic electrodes connected to non-magnetic molecules. Here we briefly refer to the key observations. OMC produced long-range impacts on ferromagnetic electrodes leading to room temperature observations of 3–7 orders current suppression.^20^ An extensive MFM study around the MTJMSD exhibited that OMC impacted the FM electrode magnetic states near the junction and created a corresponding conduction state.^16^ However, when the same OMC was connected to the non-magnetic gold and tantalum electrodes of a tunnel junction, only the current increased above the leakage level.^29–31^ Molecule's spin strongly coupled to FM electrode also yielded anomalous spin photovoltaic effect at room temperature.^28^ It is noteworthy that placing a light-sensitive molecular layer along the exposed edge of a tunnel junction with a non-magnetic gold electrode exhibited an increase in photocurrent, but the solar cell effect was not observed.^32^ We have also experimentally observed several orders of transient resistance change as a function of magnetic field on OMC-based MTJMSD.^27^ In other studies, where non-magnetic molecules were bridged between non-magnetic electrodes, above mentioned or resembling phenomenon was not observed.^33–37^ Other groups have also observed strong coupling between C_60_ molecules and ferromagnetism of the nickel electrodes leading to the Kondo splitting phenomenon without applying the estimated high magnetic field needed for this observation.^17^ In all the prior SMM based devices actual spin state was not discussed. This MCS study elaborate on the critical amount of SMMs molecular spin state necessary to observe long-range orderings.

## IV. Conclusion

We conducted a Monte Carlo simulation to study the impact of the molecular spin state (*S*_m_) on the MTJMSD and ferromagnetic electrodes. This research produced a number of lessons that help in understanding and designing futuristic molecular spintronics devices. (1) In the strong coupling regime, the molecular spin state must be above 0.2 to align two FM electrodes of an MTJMSD into an antiparallel state. (2) Magnetic susceptibility of the molecules is significantly high for the weaker molecular spin; switchable MTJMSD is only possible for a low molecular spin state. (3) In a robust ferromagnetic coupling regime, increasing molecular spin from 0–4 enabled fast equilibration and enhanced the thermal stability of molecule-induced magnetic moment. (4) Molecule-induced MTJMSD moment changed non-linearly with molecular coupling strength. (5) Magnetic electrode thickness and length are critical in determining the molecular spin state effect. With increasing, length FM electrodes started stabilizing with different phases. With increasing width, a peculiar response was observed on MTJMSD moment. MTJMSDs with some peculiar FM electrode widths behave opposite to the FM electrodes with higher and lower widths. (6) Molecule correlated magnetic phases seen in the MCS results corroborated with the experimental MFM studies on similar devices. Future studies will focus on studying the effect of spin fluctuations on MTJMSD with different molecular spin states. Our current Monte Carlo Simulation program is unable to simulate the magnetoresistance property of MTJ based on SMM. Our future work focuses on adding resistance measurement capability in different states of MTJMSDs to measure magneto resistance property.

## Author contributions

Andrew Grizzle conducted simulations studies. Andrew Grizzle developed analysis software to analyze the data, and Christopher D'Angelo wrote a C++ program under the supervision of Pawan Tyagi. José Martínez-Lillo analyzed the magnetic susceptibility experimental data. Andrew Grizzle wrote the manuscript and analyzed the data.

## Conflicts of interest

There are no conflicts to declare

## Supplementary Material

RA-011-D1RA05473B-s001
