# Supplementary material for: Spin state of a single-molecule magnet (SMM) creating long-range ordering on ferromagnetic layers of a magnetic tunnel junction – a Monte Carlo study
Source: RSC Adv. 2021 Sep 30;11(51):32275–85. doi: 10.1039/d1ra05473b (PMC9042143; doi:10.1039/d1ra05473b)
Supplement: RA-011-D1RA05473B-s001 [file RA-011-D1RA05473B-s001.pdf]

Electronic Supplementary Material (ESI) for RSC Advances.
This journal is © The Royal Society of Chemistry 2021

Supplementary Material

Spin State of Single Molecular Magnet (SMM) Creating Long Range Ordering on Ferromagnetic Layers of Magnetic Tunnel Junction -A Monte Carlo Study

Andrew Grizzle, Christopher D'Angelo, Pawan Tyagi*
Center for Nanotechnology Research and Education, Mechanical Engineering, University of the District of Columbia, Washington DC-20008, USA
Email of corresponding author: ptyagi@udc.edu

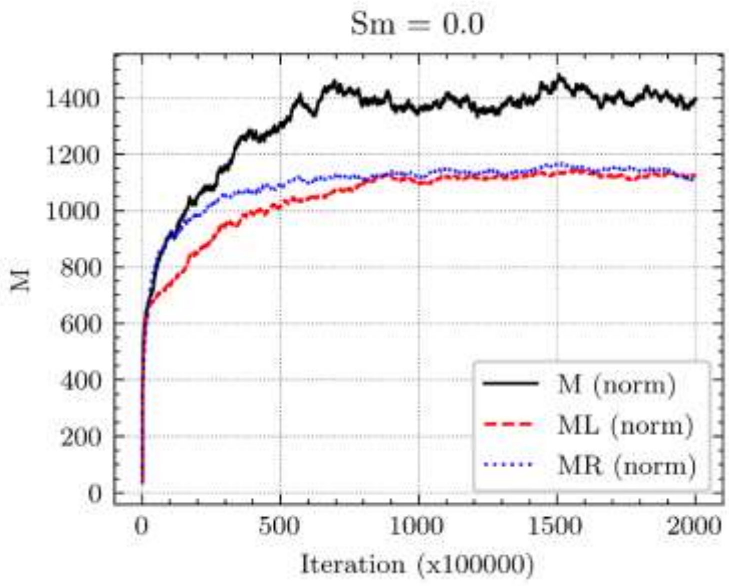

Fig. S1: M vs. time for Sm=0.

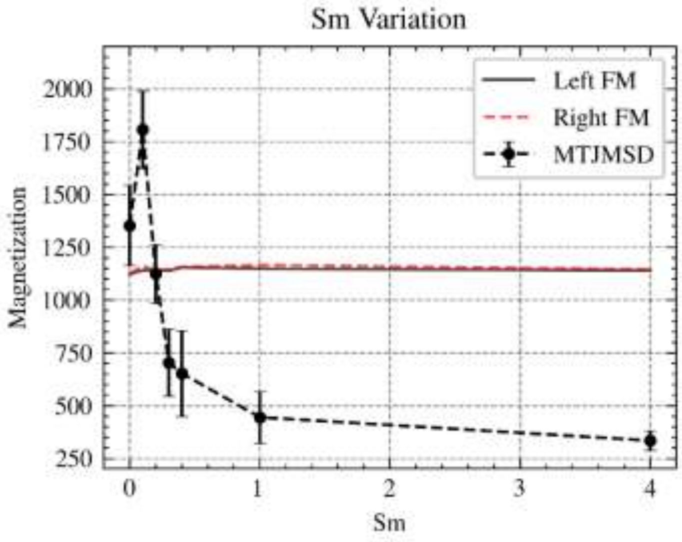

Fig.S2: Magnetization vs. Sm for 0-4 Sm range

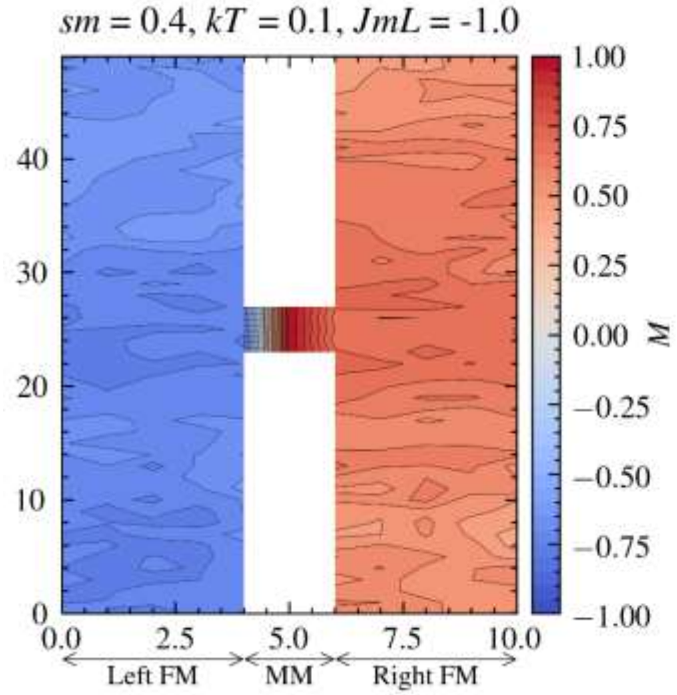

Fig. S3: Spatial correlation factor for Sm=0.4

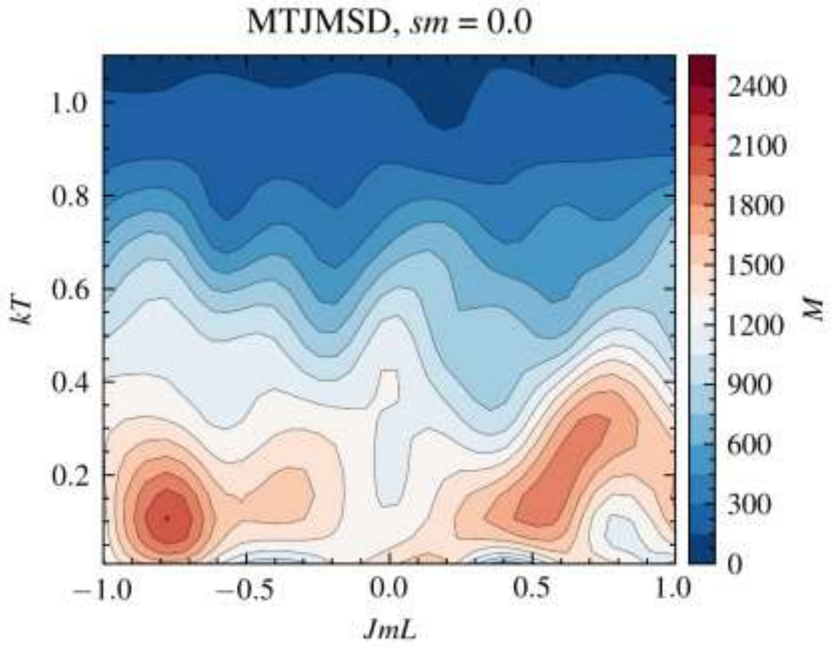

Fig. S4: kT vs JmL for Sm=0. JmR=|JmL|.
